# Is a differentiated care model needed for patients with TB? A cohort analysis of risk factors contributing to unfavourable outcomes among TB patients in two states in South India

**DOI:** 10.1186/s12889-020-09257-5

**Published:** 2020-07-24

**Authors:** Reynold Washington, Rajaram Subramanian Potty, A. Rajesham, T. Seenappa, Anil Singarajipura, Reuben Swamickan, Amar Shah, K. H. Prakash, Arin Kar, Karthikeyan Kumaraswamy, B. S. Prarthana, Bala Krishna Maryala, J. Sushma, Ramesh Dasari, Bharath Shetty, Vikas Panibatla, H. L. Mohan, Marissa Becker

**Affiliations:** 1 Karnataka Health Promotion Trust, IT Park, Rajajinagar Industrial Area, Behind KSSIDC Admin. Office, Rajajinagar, Bengaluru, Karnataka 560044 India; 2 Department of Community Health Sciences, University of Manitoba, Winnipeg, Manitoba Canada; 3 St John’s Research Institute, Bengaluru, India; 4 Office of the Joint Director (TB), Commissionerate of Health and Family Welfare, Hyderabad, Telangana India; 5 Office of the Joint Director (TB), Lady Willingdon State TB Centre, Bengaluru, Karnataka India; 6 Tuberculosis and Infectious Diseases Division, USAID/India, New Delhi, India; 7 TB Alert India, Hyderabad, India

**Keywords:** Tuberculosis, Risk-factors, Differentiated care, Unfavourable outcomes, India

## Abstract

**Background:**

TB is a preventable and treatable disease. Yet, successful treatment outcomes at desired levels are elusive in many national TB programs, including India. We aim to identify risk factors for unfavourable outcomes to TB treatment, in order to subsequently design a care model that would improve treatment outcomes among these at-risk patients.

**Methods:**

We conducted a cohort analysis among TB patients who had been recently initiated on treatment. The study was part of the internal program evaluation of a USAID-THALI project, implemented in select towns/cities of Karnataka and Telangana, south India. Community Health Workers (CHWs) under the project, used a pre-designed tool to assess TB patients for potential risks of an unfavourable outcome. CHWs followed up this cohort of patients until treatment outcomes were declared. We extracted treatment outcomes from patient’s follow-up data and from the Nikshay portal. The specific cohort of patients included in our study were those whose risk was assessed during July and September, 2018, subsequent to conceptualisation, tool finalisation and CHW training. We used bivariate and multivariate logistic regression to assess each of the individual and combined risks against unfavourable outcomes; death alone, or death, lost to follow up and treatment failure, combined as ‘unfavourable outcome’.

**Results:**

A significantly higher likelihood of death and experiencing unfavourable outcome was observed for individuals having more than one risk (AOR: 4.19; 95% CI: 2.47–7.11 for death; AOR 2.21; 95% CI: 1.56–3.12 for unfavourable outcome) or only one risk (AOR: 3.28; 95% CI: 2.11–5.10 for death; AOR 1.71; 95% CI: 1.29–2.26 for unfavourable outcome) as compared to TB patients with no identified risk. Male, a lower education status, an initial weight below the national median weight, co-existing HIV, previous history of treatment, drug-resistant TB, and regular alcohol use had significantly higher odds of death and unfavourable outcome, while age > 60 was only associated with higher odds of death.

**Conclusion:**

A rapid risk assessment at treatment initiation can identify factors that are associated with unfavourable outcomes. TB programs could intensify care and support to these patients, in order to optimise treatment outcomes among TB patients.

## Background

India contributes 27% of the global burden of Tuberculosis and 24% of Drug Resistant TB (DR-TB) and almost every third TB death globally is from India [1]. The Government of India plans to ‘End TB’ through implementation of its National Strategic Plan (NSP) - India 2017–2025. Ambitious targets for successful treatment outcomes by 2025 have been set: 92% for Drug Sensitive-(DS-TB) and 75% for DR-TB [2]. In 2017, successful treatment outcomes in India were only 79% for patients with DS-TB and 46% for those with DR-TB [3].

In India, the Revised National Tuberculosis Control Program (RNTCP) now referred to as the National TB Elimination Program (NTEP) provides free TB diagnostic and treatment services [3]. In 2012, the Government of India launched the Nikshay platform (Nik- means End, Shay means TB), a web-enabled application to facilitate monitoring of TB patients across India. All private and public health establishments were mandated to notify all TB patients who received a diagnosis or were initiated on treatment into the Nikshay. In 2018, the NTEP rolled out Poshan Abhiyan, a direct benefit cash-transfer of ₹500 per month to patients who are on treatment, in order to supplement their nutrition needs. An unsuccessful/unfavourable TB treatment outcome included the following outcomes: death, loss to follow up and treatment failure. According to the 2019 India TB report, the proportion of TB patients with an unfavourable treatment outcome was 9.3% in India, and in the southern Indian states of Telangana and Karnataka was 8.6 and 13.9%, respectively. Death was reported for 4.0% of all TB patients initiated on treatment in India, and 4.0% in Telangana and 6.2% in Karnataka [4].

In general, TB programs focus on treatment adherence in order to improve treatment outcomes. Many studies have examined factors impeding treatment adherence. The lack of adequate food, poor communication between healthcare providers and patients, beliefs in traditional healing systems, non-availability of TB services in nearby health facilities, side-effects and pill burden of the drugs, and stigma and discrimination were cited as reasons for poor adherence in qualitative studies that assessed barriers to treatment adherence [5, 6]. Understanding reasons for unfavourable treatment outcomes is critical to optimizing TB treatment programs and improving treatment success rates. There are very few studies that directly link patient level risk factors to unfavourable treatment outcomes. Studies suggest that treatment outcomes could be improved when a package of treatment adherence interventions are offered to patients on TB treatment, such as health education and counselling, digital medication monitoring, material supports to the patient, psychological support to the patient and family and staff education [7].

Worse TB treatment outcomes are often seen more frequently among individuals with co-existing conditions such as diabetes and HIV, heavy alcohol users, smokers, those aged below 25 years and above 50 years, and those who have received previous treatment for TB [8–10]. However, despite this understanding, most TB programs do not identify these individuals with these risk factors as a priority group, nor do they provide any differentiated care to these patients. The NTEP treats all TB patients similarly, with little or no differentiation in the intensity or scope of care and support to patients, with some exceptions for patients co-infected with TB and HIV and those with DR-TB. All TB patients receive routine care by TB health visitors (TBHV), who are supervised by Senior TB treatment Supervisors (STS). The NTEP field staff are minimally trained to assess and identify risks for unfavourable outcomes, or to provide individualised counselling to help the patient cope with, and overcome, treatment adherence challenges.

The United States Agency for International Aid (USAID) funded the Tuberculosis Health Action Learning Initiative (THALI), implemented by Karnataka Health Promotion Trust (KHPT), in partnership with TB Alert India and St John’s Medical College and Hospital, in three states in south India. THALI is a patient centred, family focused project which aims to enhance TB notification and treatment outcomes among vulnerable urban populations. As a part of THALI, the team designed and implemented a ‘Differentiated Care Model (DCM)’ to understand whether identifying TB patients with risks of unfavourable outcomes, and subsequently providing more intensive support where required to mitigate those risks, would result in better treatment outcomes. This paper aims to validate the hypothesis that some patients are at a higher risk of experiencing unfavourable treatment outcomes due to the presence of certain risk characteristics. We plan to evaluate the impact of the differentiated care model on improving outcomes among TB patients in a subsequent paper.

## Methods

### Study setting

The USAID/THALI project was initiated in two large cities in southern India: Bengaluru in Karnataka and Hyderabad in Telangana. In the third year of the project, the approach was refined and scaled up to other select towns and cities in Karnataka, Telangana and the neighbouring state of Andhra Pradesh. This paper focuses on analyses of patients only from Karnataka and Telangana. The selected geographies covered a total population of 18.6 million urban people in 15 districts of Karnataka and 8.1 million urban people in 6 districts of Telangana. In total, this covered 69 cities/towns (61 in Karnataka and 8 in Telangana). In these selected cities/town, the project recruited Community Health Workers (CHWs), who were local residents, to conduct outreach activities. The outreach activities included: i) awareness generation on TB; ii) referrals of symptomatic cases; iii) risk and need assessment of patients initiated on TB treatment; iv) treatment follow-ups; v) contact screening and vi) counselling services.

### Study tools

In consultation with the NTEP staff, the project developed two tools for administration to all TB patients initiated on treatment. The first tool was the “Risk and Needs Assessment (RANA)” tool and was used to identify persons with potential risks for unfavourable treatment outcomes (see additional file 1 for details). The second tool was the “Prevention Care and Support Card (PCS)” and was used to register patients for follow-up visits and record data on assessments and provision of care and support activities, test results and actions taken during each follow-up visit, until the treatment outcome was declared (see additional file 2 for details).

The CHWs were trained on how to administer the tools through both classroom and field sessions. Cluster Coordinators (CC), recruited in a ratio of 1 CC: 5 CHWs, provided on-the-job supportive supervision to the CHWs. The team pre-tested the RANA tool for 2 weeks in Bengaluru and Hyderabad, and adapted it for simplicity and uniformity in assessment, recording and interpretation of the data, before it was widely used across the project.

### Study procedure

**Fig. 1:**
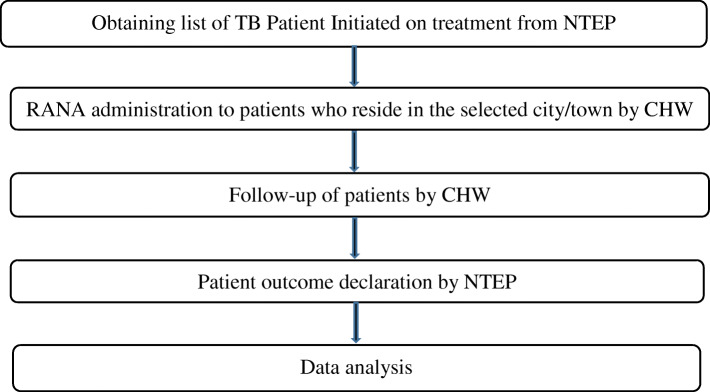
Study procedures followed

First, we obtained a list of all the persons diagnosed with TB in the project geographies from the respective NTEP staff (see Fig. 1). Subsequently, the CHWs administered the RANA tool and registered patients who consented for follow-up visits using the PCS. We could include only those TB patients who were resident within the towns/cities within the project geographies. The RANA tool was administered to the patient, however in rare instances when the patient was unable to provide the information him/herself, due to significant illness, information was collected from the primary caregiver in the family.

The RANA tool assessed the patient and/or family member’s understanding of TB and its treatment, explored family level support for the patient, listed social, nutritional and livelihood needs, identified factors that were presumed to be a risk for an unfavourable outcome to TB treatment and noted the type of follow-up preferred (in-person or other) by the patient. Each interview took approximately 25–40 min, and was conducted in a venue convenient to the patient, such as the home or the place of treatment. Initially, paper-based entries were entered onto a Management Information System, however during the course of project implementation this process changed to combine data collection and entry using a mobile application. RANA tool implementation took place in Bengaluru and Hyderabad from June, 2018.

### NTEP operational definitions

Treatment outcomes as defined by the NTEP are listed below: [11].

Cured: An individual with microbiologically confirmed TB at the beginning of the treatment who was smear or culture-negative at the end of complete treatment.

Treatment success: An individual with TB who was either cured or completed treatment.

Died: An individual with TB who was known to have died from any cause whatsoever while on treatment.

Failure: An individual with TB whose biological specimen is positive by smear or culture at the end of the treatment.

Lost to follow-up: An individual with TB whose treatment was interrupted for one consecutive month or more.

Not evaluated: An individual with TB for whom no treatment outcome is assigned (formerly “transfer out”).

Treatment regimen changed: An individual with TB who underwent a change in treatment regimen (formerly referred to as “switched over to MDR treatment”).

Unfavourable TB treatment outcomes include: Death, Failure and Lost to follow up.

### Data analysis

We combined three different data sets in order to perform our analysis. For risk identification we used data from the RANA tool. For outcome data, we used the THALI PCS tool as well as the official NTEP data from the Nikshay. The data-sets were linked using the Nikshay identity number and patient’s contact number. At the beginning of August 2019, we extracted data on patients who were 18 years or older at the time of TB diagnosis and notification, and whose RANA had been carried out in the months of July, August and September 2018. These patients had been initiated on TB treatment, 0–8 months prior to the administration of RANA, with a mean of about 2 months. We restricted the analysis to this cohort of patients in order to ensure that we had treatment outcomes for the majority of the patients. The treatment outcomes were extracted from the PCS card on July 31, 2019, or earlier. In the event that the treatment outcome data was not available in the PCS dataset, we extracted treatment outcome from the Nikshay data. In our analysis, we only included patients from Karnataka and Telangana who had data on treatment outcome declared by the month of July 2019, and who also had both a completed RANA and PCS card.

We defined two outcome indicators for the analysis: i) Death and ii) Unfavourable outcome which included death, failure or Lost to Follow Up (LFU).

Based on empirical knowledge and available evidence, we considered the following factors as potential risks for unfavourable outcomes: i) age above 60 years; ii) living alone; iii) HIV, iv) diabetes; v) undernutrition; vi) previous treatment for TB; vii) drug-resistant TB and viii) history of regular (daily) consumption of alcohol. Information on risk factors listed above were recorded based on patient’s history and/or documented laboratory reports (HIV, diabetes) as applicable.

We were unable to use BMI as our indicator of malnutrition as anthropometric measurements were not feasible within the field conditions. Hence, we used weight at the time of treatment initiation as our measure and categorised it based on whether it was below, or equal to and greater, than the median weight of TB patients as recorded in the National Guideline on Nutrition and TB (43 kg for males and 38 kg for females) [12]. We considered patients to be undernourished if their weight at the time of treatment initiation was below these values.

Data was analysed using Stata version 14. We examined socio-economic and demographic characteristics. We conducted bivariate analysis to understand whether the presence of any of the above considered risk factors were associated with the two outcome indicators. Subsequently, we applied multivariate logistic regression to determine the independent effect of each of the individual risk factors, as well as combined risk factors on the two outcomes. Thus, we considered two multivariate logistic regression models. In the first multivariate logistic regression model, we considered risk characterisation based on all the stated risks, as well as the other background characteristics of the patient. In the second model, we considered the individual risk factors along with the other background characteristics of the patient. We considered two different multivariable regression models because we wanted to understand how the individual risk factors independently influenced the outcome variables and how these risks factors as a whole influenced the outcome variables. This analysis will inform which individual characteristics that would need to be considered in the Differentiated Care Model.

### Ethical approval

The Institutional Ethics Committee of St John’s Medical College and Hospital provided the ethics approval for program data review and analysis. The State TB office and local NTEP officials in the two states provided regulatory approval for access to Nikshay data and to interview patients and conduct follow-up visits.

## Results

### Socio-demographic characteristics and risk factors

Overall, data was available for 4749 TB patients resident in the THALI project geographies within the states of Karnataka and Telangana. THALI PCS provided treatment outcome data for 4075 patients and Nikshay data was used to obtain outcomes for the remaining 674 patients. The patient’s background and risk factors are shown in Table 1. Nearly two-thirds of the patients were from Karnataka, females constituted 38% of the patient population and only 21% had completed 10th grade, 23% of patients with an initial weight measurement had a value below the median value reported for the all India level according to sex of the person (43 kg for males and 37 kg for females) and 60% had a weight that was equal to or above the median value. The initial weight was either not measured or not documented for 17% of the patients.

**Table 1:** Demographic characteristics and risk factors among TB patients

Characteristics	Percent	Number of cases
**Name of the State**		
Karnataka	66.4	3153
Telangana	33.6	1596
**Sex**		
Female	37.8	1797
Male	62.2	2952
**Initial weight**^a^		
Below median value	22.8	1081
Median value or above	60	2850
Unknown	17.2	818
**Religion**		
Hindu	70.0	3326
Muslim	26.3	1249
Others	3.7	174
**Education status**		
< 5 Standard	44.8	2126
5–10 Standard	33.9	1612
Above 10 Standard	21.3	1011
**Marital Status**		
Single	22.0	1046
Married	72.5	3443
Marriage dissolved	5.1	240
Not known	0.4	20
**Type of TB**		
Extra Pulmonary TB	24.4	1158
Pulmonary TB	75.6	3591
**Age**		
Below 60	88.0	4178
60 and above	12.0	571
**Previously treated for TB**		
No	84.0	3989
Yes	16.0	760
**DR TB**		
No	97.1	4611
Yes	2.9	138
**Drink alcohol**		
No	87.1	4135
Yes	12.9	614
**Living alone**		
No	96.1	4565
Yes	3.9	184
**HIV**		
Negative	98.2	4662
Positive	1.8	87
**Diabetes**		
No	95.1	4518
Yes	4.9	231
**Number of risks present**^b^		
No risk	58.9	2797
Only one risk present	30.0	1424
More than one risk present	11.1	528
Total percent	100	4749

Note: Included patients aged 18 years and above whose RANA was administered between July and September and also treatment outcome was declared

^a^Considered median value of 43 Kgs for males and of 38 Kgs for females

^b^Risks include, aged 60 and above, previously treated patients, DR TB patients, using alcohol, living alone, HIV positive patient, Diabetes patient

Approximately 12% of patients were aged 60 or above, 4% lived alone, 2% were reported to be HIV positive, 5% reported to have diabetes, 16% were previously treated for TB, 3% had DR-TB, and 13% reported consuming alcohol regularly. Overall, 30% of patients were identified to have only one of these stated risks, and 11% had more than one risk.

### Treatment outcomes

Treatment outcome data is shown in Table 2. In total, about 3% of patients died and 6% experienced an unfavourable outcome. A higher proportion of patients in Karnataka had an unfavourable outcome (8%). Undernourished patients experienced higher death rates and unfavourable outcome (4 and 10%), as compared to patients with initial weight equal to the national level median or higher (2 and 5%).

**Table 2:** Treatment Outcomes (death or experienced unfavourable outcomes^c^) according to background characteristics and risk factors

Characteristics	Experienced death	Experienced unfavourable outcome	Number of cases
**Name of the State**			
Karnataka	3.1	7.7	3153
Telangana	2.0	3.2	1596
**Sex**			
Female	2.1	3.6	1797
Male	3.1	7.8	2952
**Initial weight**^a^			
Below median value	4.3	10.0	1081
Median value or above	1.8	4.5	2850
Unknown	3.7	7.1	818
**Religion**			
Hindu	2.7	6.5	3326
Muslim	2.5	5.2	1249
Others	4.6	7.5	174
**Education status**			
< 5 Standard	4.0	7.7	2126
5–10 Standard	2.5	6.6	1612
Above 10 Standard	0.5	2.3	1011
**Marital Status**			
Single	1.5	5.0	1046
Married	3.0	6.4	3443
Marriage dissolved	3.8	7.9	240
Not known	0.0	10.0	20
**Type of TB**			
Extra Pulmonary TB	1.8	3.5	1158
Pulmonary TB	3	7.0	3591
**Age**			
Below 60	2.3	5.9	4178
60 and above	5.4	7.9	571
**Previously treated for TB**			
No	2.3	5.3	3989
Yes	4.9	10.8	760
**DR TB**			
No	2.6	5.9	4611
Yes	6.5	16.7	138
**Drink alcohol**			
No	2.2	5.4	4135
Yes	5.9	11.1	614
**Living alone**			
No	2.7	6.2	4565
Yes	2.2	6.0	184
**HIV**			
Negative	2.6	6.0	4662
Positive	11.5	16.1	87
**Diabetes**			
No	2.7	6.2	4518
Yes	2.2	4.8	231
**Number of risk present**^b^			
No risk	1.2	4.0	2797
Only one risk present	4.5	8.4	1424
More than one risk present	6.1	11.9	528
Total percent	2.7	6.2	4749

Note: Included patients aged 18 years and above whose RANA was administered between July and September and also treatment outcome was declared

^a^Considered median value of 43 Kgs for males and of 38 Kgs for females

^b^Risk present include, aged 60 and above, previously treated patients, DR TB patients, using alcohol, living alone, HIV positive patient, Diabetes patient

^c^Unfavourable outcome includes death, failure and LFU patients

The proportion who died or had an unfavourable outcome were higher among patients with more than one of the stated risks (6 and 12% respectively) as compared to those without any of the stated risks (1% death and 4% unfavourable outcome). Similarly, patients having only one of the stated risks also had experienced higher deaths (5%) and unfavourable outcomes (8%) than those without any risk.

Table 3 shows the results using logistic regression to determine the odds ratios and adjusted odds ratios of the patient experiencing death, or unfavourable outcome as defined earlier, according to background characteristics and any risk. Patient with more than one of the stated risk factors had a significantly higher likelihood of dying and/or experiencing an unfavourable outcome as compared to patients without any of the risk factors (AOR: 4.19; 95%CI: 2.47–7.11 for death; AOR: 2.21; 95% CI: 1.56–3.12 for unfavourable outcome). Undernutrition and education status below matriculation (10th grade) of the patient were also seen to significantly result in death and/or unfavourable outcome. TB patients with initial weight below the median weight were 2.1 times (95%CI: 1.38–3.14) and 2.0 times (95%CI: 1.50–2.61) more likely to die or experience unfavourable outcome respectively, as compared to patients with initial weight equal to the median or higher. Patients from Karnataka also had a significantly higher likelihood of an unfavourable outcome as compared to patients from Telangana (AOR: 2.35; 95%CI: 1.71–3.23) as did males as compared to females (AOR: 1.63; 95% CI: 1.20–2.21).

**Table 3:** Multivariate logistic regression for treatment outcomes (death and unfavourable outcomes) that considered background characteristics and combined risk of DCM

Characteristics	Experienced death					Experienced unfavourable outcome^c^				
	UOR	AOR	95% CI		p- value	UOR	AOR	95% CI		p- value
**Name of the State**										
Telangana (Reference)	1.00	1.00				1.00	1.00			
Karnataka	1.55	1.42	0.94	2.16	0.099	2.52	2.35	1.71	3.23	< 0.001
**Sex**										
Female (Reference)	1.00	1.00				1.00	1.00			
Male	1.53	0.97	0.64	1.48	0.883	2.28	1.63	1.20	2.21	0.002
**Initial weight**^a^										
Median value or above (Reference)	1.00	1.00				1.00	1.00			
Below median value	2.45	2.08	1.38	3.14	< 0.001	2.38	1.98	1.50	2.61	< 0.001
Weight unknown	2.05	2.12	1.32	3.38	0.002	1.64	1.74	1.25	2.43	0.001
**Religion**										
Muslims (Reference)	1.00	1.00				1.00	1.00			
Hindus	1.09	0.97	0.64	1.48	0.888	1.26	1.09	0.81	1.46	0.573
Others	1.89	1.84	0.81	4.15	0.143	1.47	1.44	0.76	2.72	0.261
**Education status**										
Above 10 Standard (Reference)	1.00	1.00				1.00	1.00			
< 5 Standard	8.28	5.39	2.11	13.79	< 0.001	3.59	2.68	1.67	4.31	< 0.001
5–10 Standard	5.12	3.96	1.53	10.22	0.004	3.02	2.41	1.50	3.86	< 0.001
**Marital Status**										
Single (Reference)	1.00	1.00				1.00	1.00			
Married	2.01	1.06	0.60	1.87	0.839	1.30	0.88	0.63	1.24	0.472
Marriage dissolved	2.51	0.93	0.38	2.26	0.872	1.64	1.07	0.59	1.94	0.815
Not known	NE	NE	NE	NE	NE	2.12	2.67	0.57	12.44	0.211
**Type of TB**										
Extra Pulmonary TB (Reference)	1.00	1.00				1.00	1.00			
Pulmonary TB	1.68	1.00	0.99	0.60	1.632	0.98	1.31	0.92	1.86	0.138
**Number of risk present**^b^										
No risk (Reference)	1.00	1.00				1.00	1.00			
Only one risk present	3.94	3.28	2.11	5.10	< 0.001	2.21	1.71	1.29	2.26	< 0.001
More than one risk present	5.40	4.19	2.47	7.11	< 0.001	3.28	2.21	1.56	3.12	< 0.001

Note: Included patients aged 18 years and above whose RANA was administered between July and September and also treatment outcome was declared. UOR – Unadjusted odds ratio. AOR – Adjusted odds ratio. CI – Confidence Interval

^a^Considered median value of 43 Kgs for males and of 38 Kgs for females

^b^Risk include, aged 60 and above, previously treated patients, DR TB patients, using alcohol, living alone, HIV positive patient, Diabetes patient

^c^Unfavourable outcome includes death, failure and LFU patients

Results from the second logistic regression that considered the stated risk factors individually are given in Table 4. Out of the stated risk factors considered, four factors including patients who are aged 60 and above (AOR: 2.15; 95%CI: 1.37–3.37), who consume alcohol regularly (AOR: 2.09; 95%CI: 1.35–3.25), who were previously treated for TB (AOR: 1.65; 95%CI: 1.08–2.51), and those who were living with HIV (AOR: 4.75; 95%CI: 2.29–9.86) were significantly more likely to experience death, as compared to patients without these risks. Age of the patient was not a significant risk factor for an unfavourable outcome, though it was for death. Additionally, patients with DR-TB were significantly more likely to experience an unfavourable outcome as compared to patients without DR-TB (AOR: 2.33; 95%CI: 1.41–3.87). Those patients with undernutrition were found to have a significantly higher likelihood of death (AOR: 1.98; 95%CI: 1.30–3.00) and/or unfavourable outcome (AOR: 1.89; 95%CI: 1.43–2.50).

**Table 4:** Multivariate logistic regression for (death and unfavourable outcomes) that considered background characteristics and individual risks of DCM

Characteristics	Experienced death					Experienced unfavourable outcome^b^				
	UOR	AOR	95% CI		p- value	UOR	AOR	95% CI		p- value
**Name of the State**										
Telangana (Reference)	1.00	1.00				1.00	1.00			
Karnataka	1.55	1.54	1.01	2.35	0.045	2.52	2.46	1.79	3.39	< 0.001
**Sex**										
Female (Reference)	1.00	1.00				1.00	1.00			
Male	1.53	0.95	0.61	1.48	0.825	2.28	1.69	1.24	2.30	0.001
**Initial weight**^a^										
Median value or above (Reference)	1.00	1.00				1.00	1.00			
Below median value	2.45	1.98	1.30	3.00	0.001	2.38	1.89	1.43	2.50	< 0.001
Weight unknown	2.05	1.96	1.22	3.15	0.005	1.64	1.70	1.22	2.37	0.002
**Religion**										
Muslims (Reference)	1.00	1.00				1.00	1.00			
Hindus	1.09	0.94	0.62	1.45	0.788	1.26	1.10	0.82	1.47	0.544
Others	1.89	1.71	0.75	3.91	0.205	1.47	1.40	0.74	2.66	0.304
**Education status**										
Above 10 Standard (Reference)	1.00	1.00				1.00	1.00			
< 5 Standard	8.28	5.38	2.10	13.83	< 0.001	3.59	2.74	1.71	4.41	< 0.001
5–10 Standard	5.12	3.99	1.55	10.31	0.004	3.02	2.41	1.51	3.87	< 0.001
**Marital Status**										
Single (Reference)	1.00	1.00				1.00	1.00			
Married	2.01	1.09	0.62	1.93	0.763	1.30	0.95	0.68	1.34	0.779
Marriage dissolved	2.51	0.88	0.35	2.23	0.795	1.64	1.22	0.66	2.24	0.523
Not known	NE	NE	NE	NE	NE	2.12	3.23	0.70	14.96	0.133
**Type of TB**										
Extra Pulmonary TB (Reference)	1.00	1.00				1.00	1.00			
Pulmonary TB	1.68	1.09	0.66	1.80	0.737	2.06	1.37	0.96	1.95	0.081
**Age**										
Below 60 (Reference)	1.00	1.00				1.00	1.00			
60 and above	2.39	2.15	1.37	3.37	0.001	1.36	1.12	0.79	1.60	0.533
**Previously treated for TB**										
No (Reference)	1.00	1.00				1.00	1.00			
Yes	2.17	1.65	1.08	2.51	0.020	1.58	1.18	2.11	0.00	0.003
**DR TB**										
No (Reference)	1.00	1.00				1.00	1.00			
Yes	2.61	1.83	0.86	3.89	0.117	3.22	2.33	1.41	3.87	0.001
**Drink alcohol**										
No (Reference)	1.00	1.00				1.00	1.00			
Yes	2.71	2.09	1.35	3.25	0.001	2.16	1.38	1.01	1.88	0.043
**Living alone**										
No (Reference)	1.00	1.00				1.00	1.00			
Yes	0.79	0.68	0.24	1.89	0.460	0.75	0.40	1.42	0.38	0.473
**HIV**										
Negative (Reference)	1.00	1.00				1.00	1.00			
Positive	4.96	4.75	2.29	9.86	< 0.001	3.01	2.61	1.41	4.82	0.002
**Diabetes**										
No (Reference)	1.00	1.00				1.00	1.00			
Yes	4.96	0.74	0.29	1.86	0.521	0.75	0.70	0.37	1.32	0.266

Note: Included patients aged 18 years and above whose RANA was administered between July and September and also treatment outcome was declared. UOR – Unadjusted odds ratio. AOR – Adjusted odds ratio. CI – Confidence Interval. NE – Not estimated

^a^Considered median value of 43 Kgs for males and of 38 Kgs for females

^b^Unfavourable outcome includes death, failure and LFU patients

When we considered all the stated risk factors individually into the model, patients from Karnataka experienced significantly higher likelihood of death (AOR: 1.55; 95%CI: 1.01–2.35) as well as unfavourable outcome (AOR: 2.46; 95%CI: 1.79–3.39) as compared to patients from Telangana. Educational status above 10th standard was found protective against both death as well as unfavourable outcome in comparison with those with lesser education. The odds of males experiencing an unfavourable outcome was 1.7 times (95%CI: 1.24–2.30) higher than females.

## Discussion

The identification of patients at high risk of experiencing an unfavourable outcomes is essential in order to ensure that these high-risk patients are provided with a more intensified follow up and this is an essential first step in a “differentiated care model”. In our study, we found that those educated less than 10th standard, living with HIV, undernourished, with a history of previous TB treatment, with DR-TB, with regular alcohol use and age > 60 years were more likely to die as a result of TB. With the exception of age, all of these factors also had a higher likelihood of an unfavourable outcome. Additionally, males and those from Karnataka also has a higher risk of unfavourable outcome. All of these risk categories would therefore qualify for a more intensive model of care and support during TB treatment.

The differentiated care model is a well-known approach for care for people living with HIV (PLHIV). This model has been successfully used to categorise stable versus unstable PLHIV in order to provide differentiated care [13]. In India, PLHIV with TB are: screened regularly using a four symptom screening, a priority group for diagnosis by Cartridge based Nucleic Acid Amplification Test (CB-NAAT), and recommended for early initiation of both anti-tuberculosis treatment (ATT) as well as early initiation of anti-retroviral treatment (ART). Differentiated care is provided by ART centres, which are staffed by a multidisciplinary team of doctors, counsellors, nurses, pharmacists and others. Similarly, for DR-TB, the Government of India has established a wide network of labs across the country to enable early diagnosis and has created nodal treatment centres in almost every district, staffed by doctors, laboratory technicians and counsellors. Thus, TB patients with HIV or DR-TB have greater access to more rapid, individualised and more comprehensive care, from multi-skilled teams. We have shown in our study that there are at least 4–5 other categories of patients that also require intensive and personalised care from a team of care providers.

We found that only five of the risk factors that we had initially considered, were significantly increased the likelihood of death and/or unfavourable outcomes. Diabetes and living alone did not turn out to be significantly associated with an increased odds of death or with unfavourable outcome. However, undernutrition was identified as an independent risk factor for death and/or unfavourable outcomes. We intended to use body mass index as the measure for malnutrition. However, measuring weight and height were not always feasible to do in the field. Moreover, for those who did have data available on weight and height, these were often collected at different times during the course of the disease and its treatment. Hence, we chose the single indicator of whether the initial weight that was measured was lower, or equal to and greater than the median, as referred to in the handbook on Nutrition and TB. While this may be a very crude measure, it is fairly simple and straightforward for front line workers to collect and to manage.

A systematic review indicated diabetes to be associated with death and unfavourable outcome among those on TB treatment [14]. This association was not seen in our study. This could be due to the fact that diabetes screening among TB patients in our project geographies is not universal and was captured only from available patient reports. The prevalence of diabetes in the general population ranges from 12 to 18%, while in our study, it was less than 5% among TB patients. Understanding the true prevalence of diabetes among people with TB is important for appropriate management and is recommended within the Ayushman Bharat primary health care program in India [15]. The incidence of peripheral neuropathy in patients with TB and diabetes tends to be high [16] but this can be managed when identified during intensive follow-up. Hence, identification of diabetes among TB patients and provision of relevant care would be important in a differentiated care model, even though diabetes did not stand out significantly in our analysis.

Age above 60 years was not independently associated with an unfavourable TB treatment outcome in the multifactorial logistic regression analysis, though it was significantly associated with higher rates of death. The risk of death is high and therefore these patients require additional supports. Perhaps, TB death in this age is confounded by other co-morbidities. This needs further exploration. We therefore feel that we still need to include age above 60 years within the differentiated care model, because these TB patients may require more intensive care and support, in order to prevent untimely death, whether directly as a result of TB or because of other underlying disease conditions, which should be adequately screened for and appropriately managed. The risk of unfavourable outcomes appears to be higher in Karnataka, when compared to the neighbouring states. This reasons for this difference needs further exploration.

In many resource limited settings, the provision of follow up comprehensive care for all TB patients is often not feasible, nor necessary. However, identifying and ensuring that all patients with one or more risks associated with experiencing any unfavourable outcome or death alone are followed up intensively and are provided with a differentiated care approach, that addresses their specific treatment-related, social, nutritional, behavioural and psychological needs.

TB patients have diverse disease, demographic and behavioural characteristics. These characteristics are easily obtained through patient history and, in India, are expected to be recorded for TB patients on the Nikshay platform. Therefore, identifying patients with these risk factors is feasible in India, if a risk and needs assessment is carried out and documented into Nikshay in a systematic manner. Data analytics can be directly built into the system to highlight a patient who needs the differentiated care approach. The study has indicated that few of the risks that are important for assessment of whether a TB patient requires more specific and comprehensive care or whether routine care is sufficient. The fields for all these risk factors are configured on the current Nikshay platform, but are rarely given adequate attention.

Some of the limitations of our study include the following. Previous studies have indicated age below 25 years as a risk factor for poor outcomes but our analysis was restricted to those above the age of 18 and the number between 18 and 25 was not large enough to analyse separately. In a few instances, we noted inconsistency between treatment outcomes reported in Nikshay and our data. In these instances, we used our recorded outcome as we were able to validate this directly from the patient or a family member. For 14% of patients, we depended on Nikshay, as we did not have an outcome recorded in our database. The characteristics of this group of patients for whom we did not have an outcome recorded in the PCS, were not independently analysed to examine whether any differences existed between this group and those for whom we had treatment outcomes recorded in the PCS. Finally, since the RANA administration was carried out on an average of almost 2 months after the initiation of treatment, there could be underestimation of deaths, as about 50% of TB deaths occur within 2 months of treatment initiation [17]. Our data was also unable to draw inferences on patient’s treatment adherence during the follow-up period. However, we note that reports of missed doses among patients that were intensively followed up by CHWs was rare.

## Conclusions

Achieving favourable treatment outcomes for drug sensitive TB to 92% and DR-TB to 75% requires innovative approaches. The Differentiated Care Model (DCM), with an initial Risk and Needs Assessment (RANA), appears to be an essential first step innovation, in order to first identify those at greater risk of an unfavourable treatment outcome and then to ensure follow up care and support tailored to their needs. The study provides a model, with process and tools to implement this innovation at scale.

## Supplementary information

**Additional file 1.** Tool for risk and needs assessment (RANA).

**Additional file 2.** Prevention Care and Support Card (PCS).

## Data Availability

Some restrictions will apply with sharing the data. Data cannot be shared publicly without the approval form the donor agency and the concerned NTEP office. The de-identified data used for the paper can be obtained from the corresponding author with permission from KHPT, the donor agency and the concerned State TB officer.

## Abbreviations

AOR: Adjusted odds ratio
ART: Anti-retroviral treatment
ATT: Anti-tuberculosis treatment
CB-NAAT: Cartridge based nucleic acid amplification test
CC: Cluster coordinators
CHW: Community health workers
CI: Confidence interval
DCM: Differentiated care model
DOTS: Directly observed treatment short-course
DR-TB: Drug resistant TB
DS-TB: Drug sensitive TB
HIV: Human immunodeficiency virus
LFU: Lost to Follow-up
NSP: National strategic plan
NTEP: National TB Elimination Program
PCS: Prevention care and support card
PLHIV: People living with HIV
RANA: Risk and needs assessment
RNTCP: Revised National Tuberculosis Control Program
STS: senIor TB treatment supervisors
TB: Tuberculosis
TBHV: TB health visitor
THALI: Tuberculosis health action learning initiative
USAID: United States Agency for International Aid
