# Supplementary material for: Is a differentiated care model needed for patients with TB? A cohort analysis of risk factors contributing to unfavourable outcomes among TB patients in two states in South India
Source: BMC Public Health. 2020 Jul 24;20:1158. doi: 10.1186/s12889-020-09257-5 (PMC7379762; doi:10.1186/s12889-020-09257-5)
Supplement: Supplementary file 1 — Additional file 1. Tool for risk and needs assessment (RANA). [file 12889_2020_9257_MOESM1_ESM.pdf]

Risk Assessment and Needs Assessment *Date:*

Nikshay Id DMC Name: TU Name:

Facility Name PRAD Id:

Name:……………………………………………………..<br>Current Address.…………………………….….<br>………………………………………………………..<br>……………………………………………………….<br>Land Mark:………………………………………….<br>permanent Address ……………………………….<br>……………………………………………………….<br>……………………………………………………….										**Age:** ……Yrs **DOB:** DD/MM/YYYY<br>**Gender:** M F TG<br>**Religion:** Hindu Muslim<br>Christian Other<br>**Education: Education:** Cannot Read/Write <5 std 5-10 std. 12th Diploma Graduate Post Graduate<br>**Marital Status:** Single Married<br>Widowed separated/divorced<br>**Occupation:**……………
Contact No										
Alternate No										

Type of TB: Pulmonary Extra Pulmonary

History of TB treatment: Never treated or treatment <1 month Previously treated for TB

Anti TB medication from: Private sector Public sector

Treatment Regimen Initiated: DS TB DRTB

Date of treatment initiation: DD/MM/YYYY

Risk assessment

Risk factor	Assess if present (Y/N)	Action Taken (Y/N)	Details of Action taken
**About TB**			
**Does patient understand TB disease and/or treatment?**<br>*Do you know what disease you have? what did the health worker say to you?*<br>*How does TB spread? By air / Droplet*<br>*What is the common symptom of TB? Cough*<br>*What is the test for lung TB? Sputum test*<br>*Is TB Curable? Yes TB is curable*			
**Acceptance of TB disease and/or treatment**<br>*Does the patient accept that s/he has TB?*<br>*Is the patient ready to take treatment for long term (At least 6 months)?*<br>*Is the patient willing to take tablets and / or injection as prescribed?*			
**About Person**			
Regular travel/ likely migration			
Co-existing conditions (write codes)<br>*a. HIV b. DM c. Silicosis d. Undernutrition e. Pregnancy f. Breast feeding G. Other Specify*			
Alcoholism			
Tobacco Addiction			

Have you ever experienced discrimination or denial of rights because of TB?			
**About family**			
Living Alone / No Care Giver			
Do you have the support of family members?			
Is there some body who knows about your TB status who can support you in treatment?			
Did you face any crisis recently in the family or any plan of social event in near future?			
Financial difficulties			
**About treatment**			
Problem with access to drugs			
Were you irregular to treatment previously?			
Symptoms persisting			

Social security needs Assessment

**Social entitlements**	**Specify Details**	**Have this? Y/N/NA**	**Need support for availing this? Y/N**	**If linked through program, date of linkage**
	Aadhar Card			
	Bank Account			
	Ration Card (APL/ BPL)			
	DBT linkage			
	Link to free TB medicines			
Nutrition support	Anthyodaya			
	ICDS			
	Others			
Livelihood support	Income generation activity			
	Pension schemes			
Health related schemes (Health Insurance etc)	Janani Suraksha Yojana (JSY)			
	Rashtriya Swasthya Bima Yojana(RSBY)			
	Other state related health schemes			
	Other Health Insurance Schemes			
Any other schemes				

Risk Assessment and Needs Assessment *Date:*

**Patients preferred mode of care and support**		
In Person<br>THALI RNTCP<br>JEET<br>Support Group	TB Careline<br>Technology based support (99 DOTS, Merm Etc).<br>Call centre	Facility / provider Support<br>Self-monitored/ Family supported only<br>Other (Specify) ……..………
I …………….agree to this option/s for follow up Signature of the patient:		

Risk and Needs Assessment done by

Project: THALI JEET RNTCP

Name: __________________ Designation:

staff Id:…………………………………………..
