# Supplementary material for: Is a differentiated care model needed for patients with TB? A cohort analysis of risk factors contributing to unfavourable outcomes among TB patients in two states in South India
Source: BMC Public Health. 2020 Jul 24;20:1158. doi: 10.1186/s12889-020-09257-5 (PMC7379762; doi:10.1186/s12889-020-09257-5)
Supplement: Supplementary file 2 — Additional file 2. Prevention Care and Support Card (PCS). [file 12889_2020_9257_MOESM2_ESM.pdf]

Date of PCS registration: DD/MM/YYYY

PREVENTION CARE & SUPPORT CARD

PRAD ID ………………………Nikshay Id:………………

PCS ID: **Staff Name & Code** **Supervisor Name**

1. Patient Registration Details

Name:
Gender: ☐ M ☐ F ☐ TG
Age: …… Yrs, Date of Birth:
Current Address:

Town:
Land Mark:
Permanent Address:

TU Name:
DMC Name:

Occupation:

Education: ☐ Cannot Read/Write ☐ <5 std ☐ 5-10 std. ☐ 12th ☐ Diploma ☐ Graduate ☐ Post Graduate

Religion: ☐ Hindu ☐ Muslim ☐ Christian ☐ Other Specify-----

Marital Status: ☐Single ☐Married ☐Widowed ☐separated/divorced

Aadhaar No:

Contact No:
Care Giver Name: Relationship to Patient:
Care giver's Contact No:

Treatment Supporter Name:

Treatment Supporter Contact no :

Risk present ☐No ☐Yes
Specify risk

2.Comorbidity and Treatment Details

Co-existing Conditions
HIV Status: ☐Unknown ☐ Positive ☐ Negative
Initiated on ART: ☐ Yes ☐ No

Diabetes Status: ☐Unknown ☐Yes ☐No
If yes is patient on diabetes treatment ☐ Yes ☐No

Hb:%........gm% ☐ Not Available

Other conditions Specify………………………

For women aged 15-49yr
On Oral Contraceptive Pills ☐ Yes ☐ No

Currently pregnant/ breast feeding
☐ Yes ☐ No

Basis of TB diagnosis
☐Microbiologically Confirmed
- ☐ Microscopy
- ☐ CBNAAT/LPA
- ☐ Culture & Sensitivity

☐Clinically Diagnosed
Type of TB :
☐ Pulmonary TB
☐ Extra Pulmonary TB

Nutritional Assessment at treatment initiation
Weight ……Kgs Height ……Cms BMI ……...Kg/m^2
MUAC….. ……Cms (child <5 yrs, Pregnant women)

Treatment details

History of TB treatment: ☐New ☐Previously treated for TB

Anti TB medication from ☐Private sector ☐Public sector

Name of the Health Care facility which initiated treatment:

Treatment Regimen Initiated: ☐DS TB ☐DR TB

Receiving FDC: ☐ Yes ☐ No
Date of treatment initiation DD/ MM/ YY__Date of initiation of CP: DD/MM/YY

3. Contact Screening

Date Of screening	DD/MM/YY		DD/MM/YY		DD/MM/YY	
	<6 yrs	>6yrs	<6 yrs	>6yrs	<6 yrs	>6yrs
No of household contacts						
No screened						
No with Symptoms						
No referred for test/doctor						
No Diagnosed						
No put on treatment						

Children Less than 6 years given IPT								
Name of child	Wt (Kg)	Dose (Mg)	M 1	M 2	M 3	M 4	M 5	M6

Details of contacts referred through contact tracing					
Name of contact	Age	Gender	Relationship to index patient (code)	PRAD ID	TB Y/N

4. Patient Follow Up Visit Details

SI no	Contact date	Mode of contact (Code)	Person Contacted (Code)	Have you missed day TB Medicine (Y/N)	How many days of TB medicine have you Missed?	Reason for Missing doses	Symptom (code)	Risk factors (Code)	Needs Assessed (Code)	Action Taken (code)	Record Weight in kg	MUAC for children <5yrs, pregnant women in Cm	Remarks
1													
2													
3													
4													
5													
6													
7													
8													
9													
10													
11													
12													
13													
14													
15													
16													
17													
18													

Follow up lab test

Date of Follow up test	Type of test done.	Result

Patient Treatment outcome (after discussion with HCP)

☐ Cured ☐ Treatment completed ☐ Not Evaluated ☐ Loss to follow up ☐ Failure ☐ Regimen Change ☐ Death

☐ Others (specify) ……………………… ☐ ORW unable to follow up (reason) ……………………………………..

New PRAD ID if applicable…………………………………….. Outcome declaration date DD/MM/YYYY

5. Post Treatment Completion Follow Up *- once in 6 months till 2 years after completion of treatment*

Follow up due date	Actual visit / phone date	Mode of contact (Code)	General condition as reported by patient/ Family (Code)	Comorbid condition diagnosed if any (Code)	Weight in Kg (if available)	Referral for TB testing Y/N	PRAD ID if Y	Any contact has TB symptoms (Y/N)	PRAD ID if Y	Remarks
